# Applying Automated Artificial Intelligence Models on Lateral Cephalometric Parameters to Accurately Classify Arab Orthodontic Patient Patterns

**DOI:** 10.1002/cre2.70372

**Published:** 2026-06-07

**Authors:** Kareem Midlej, Peter Proff, Nezar Watted, Fuad A. Iraqi

**Affiliations:** ^1^ Department of Clinical Microbiology and Immunology, Gray Faculty of Medicine and Health Sciences Tel Aviv University Tel Aviv Israel; ^2^ Department of Orthodontics, University Hospital of Regensburg University of Regensburg Regensburg Germany; ^3^ Center for Dentistry Research and Aesthetics Jatt Israel; ^4^ Department of Orthodontics, Faculty of Dentistry Arab American University Jenin Palestine

**Keywords:** cephalometric parameters, deep‐learning, diagnosis, machine‐learning, personalized medicine, skeletal malocclusion

## Abstract

**Objectives:**

Malocclusion and the treatment process can affect the person's quality of life, which includes physical and psychological effects. Classifying the patient accurately is critical to achieving the desired output. This study aimed to improve the current diagnostic process with artificial‐intelligence algorithms using the lateral cephalogram.

**Materials and Methods:**

The study sample consisted of 1014 Arab patients diagnosed with skeletal class I, II, or III. In this study, we used linear discriminant analysis (LDA), random forest (RF), decision tree (DT), K‐nearest neighbors (KNN), support vector machine (SVM), and naive Bayes (NB) as classification models. In addition, we calculated the parameters' importance using two techniques—the impurity decreases and the leave‐one‐feature‐out (LOFO) technique. Finally, we applied an artificial neural network (ANN) to classify the patients accurately.

**Results:**

One of the influential models presented in this study was the model that included only the parameters Wits appraisal, SNB, SNA, and ML‐NSL angles. It could classify the patients with an accuracy of 0.98. In addition, we applied the leave‐one‐feature‐out technique (LOFO) for multiple random forest models and found that the Calculated_ANB (ANB angle‐individualized ANB) and Wits appraisal were the most important parameters in the random forest models. Besides, age and gender were in 8th and 21st places (out of 26 variables). Furthermore, the decision tree results demonstrated the distinct characteristics of this ethnic group, which were presented by different ranges of ANB angles that define every skeletal class. The results showed that the tree's root classified the patient as skeletal class III when the ANB angle is less than 0.084 degrees, and skeletal class II if the ANB angle is greater than 1.23.

**Conclusions:**

In summary, this research presented a model enabling orthodontists to precisely classify orthodontic patients. Further research should include different ethnic groups to validate our findings.

## 1 Introduction

Malocclusion conditions and treatment can significantly affect a person's life. A previous study by Zhang et al. (2006), which examined the effect of malocclusion on quality of life, demonstrated that malocclusion and its treatment can affect physical health regarding pain, speech, and mastication. In addition, malocclusion can affect the psychological health (self‐concept) and social status (perceived attractiveness by others, social acceptance, and perceived intelligence). Previous studies showed that malocclusion is a complicated disorder produced by the combination of multiple factors, such as genetics, environment, ethnic factors, nonnutritive sucking habits, impaired nasal breathing, and functional atrophy of the maxilla (Zohud et al. 2023; Katz et al. 2004; Peres et al. 2007; Heimer et al. 2008; Sousa et al. 2014). Research examining the association between oral habits, mouth breathing, and malocclusion, and a cross‐sectional study on 3017 children, found that increased bad habits and mouth breathing were associated with more severe malocclusions. In addition, a significant association was found between bad habits and increased overjet and open bite (Grippaudo et al. 2016).

The lateral cephalogram is the most commonly used method to analyze malocclusion. Anatomic landmarks are identified, and linear and angular measurements are used to analyze the patient's malocclusion (Kim and Lee 2021). Cephalometric images, which were introduced first by Broadbent in 1931, demonstrate important diagnostic information about the relationship between skeletal and dental structures (Broadbent 1931). Research that assessed the influence of lateral cephalometric radiography in orthodontic diagnosis and treatment planning found that the majority of Portuguese orthodontists judge that the lateral cephalogram is important to producing a treatment plan, but it doesn't seem to have an influence on orthodontic treatment planning (Durão et al. 2015). The skeletal class, defining the sagittal relationship between the mandibula and maxilla, can be classified by the ANB angle and the Wits appraisal (Jacobson 1975). Skeletal classes II or III are characterized by a dysgnathic relation between the two jaws, while skeletal class I is characterized by a neurobasal relation of maxilla and mandible (Paddenberg et al. 2023). Over the years, different approaches have been applied to diagnose patients with skeletal malocclusion. Cephalometric parameters have been used to develop individualized ANB angles or Wits appraisal based on specific population data equations. For example, Panagiotidis and Witt (Panagiotidis and Witt 1977) study in 1977, created an equation for the individualized ANB as ANBind = (−35.16 + 0.4*SNA + 0.2*ML‐NSL). Recently, Paddenberg et al. (2023) established an improved regression formula for individualized ANB angle and Wits appraisal that can improve the assessment of sagittal skeletal class in clinical orthodontic practice, and reached *R* ^2^ = 0.97 in the formula: Wits_ind_ = 57.510 + 1.526ANB‐0.634SNA‐0.666SN‐Occl. Despite the enhanced result gained from the updated formula established by Paddenberg et al. (2023) and others, these formulas still have major limitations, as they were based on specific populations only. In some studies, the equations were based on a relatively small sample size. Over the years, many equations have emerged and been used by orthodontics to make non‐uniformity diagnosis approaches.

With the development of deep learning and computer vision, recent medical studies have demonstrated the practical value of combining computer science, statistics, and medical problem use cases (Tafala et al. 2022). The four major AI‐driven dentistry fields are classification, most commonly used, followed by regression, detection, and segmentation (Liu et al. 2021). According to a recent review that was done by Nordblom et al. (2024), which aimed at appraising hot topics in the field of artificial intelligence (AI) in orthodontics, one of the most researched missions in orthodontics is automated lateral cephalogram landmark detection. Specific parameters are deduced and employed from detected landmarks for orthodontic treatment planning and evaluation. In a study that aimed to provide an accurate skeletal diagnostic system by incorporating a convolutional neural network (CNN) diagnostic system with lateral 5890 cephalograms and demographic data. The resulting system exhibited > 90% accuracy for vertical and sagittal skeletal diagnosis while eliminating the landmark detection process (Yu et al. 2020). In a study that was performed on German patients skeletal class I, and II, and aimed mainly to develop a machine‐learning model to classify orthodontic patients as skeletal class I or II based on minimal cephalometric parameters, found that the KNN model with the variables ANB angle, Wits appraisal, and SNB angle, demonstrated sufficient accuracy of 90.53% (Paddenberg‐Schubert et al. 2025a), while in a study that examined the ability to classify German orthodontic patients as skeletal class I or III, found that SNA, SNB, and ML‐NSL angles were able to predict the classification as skeletal class I or III in the GLM model gained an accuracy of 99% (Paddenberg‐Schubert et al. 2025b). A recent study that was performed by Midlej et al. (2024), examined the ability of machine‐learning models to classify skeletal class II and III Arab orthodontic patients accurately and found that the support vector machine (SVM) model with the parameters Wits appraisal and SNB angle as input, was able to predict the allocation of patients to either skeletal class II or III with an accuracy of 95%, compared to a value of 99% when 26 parameters (24 cephalometric parameters and two covariates—gender and age) were used. One major challenge in current AI research in orthodontics is limited generalizability. Besides, comparing AI across different studies is difficult since outcomes and outcome metrics vary widely (Nordblom et al. 2024).

To our knowledge, the research presented in this study is the first to be conducted on a cohort of 1014 Arab residents of Israel who were classified as having skeletal occlusion (class I) or malocclusions (classes II and III). Therefore, the main aim of this study was to derive novel deep‐learning and machine‐learning models based on the most important parameters to gain substantially higher accuracy in the classification of skeletal classes I, II, or III. We applied various AI models and input variables to detect the best‐fitting models.

## 2 Materials and Methods

### 2.1 Ethical Statement

Data collection of the present study was in line with the guidelines and followed the regulations of the Ethics Committee of Tel‐Aviv University, approval number (0010557‐4). In addition, this study conformed to the Strengthening the Reporting of Observational Studies in Epidemiology (STROBE) guidelines. All patients who participated in this study were assessed at the Orthodontic Research Center based in Jatt, Israel. Patients aged 18 years or older or parents/guardians younger than 18 agreed to participate in this quantitative, observational study after receiving detailed information and signing a corresponding informed consent form. This sample consisted of the coded records of 1014 patients diagnosed as skeletal class I, II, or III. All data were collected as part of the routine orthodontic diagnostics and standard of care, which had been taken for orthodontic treatment only. The research sample consisted of Arab orthodontic patients with skeletal class I (*n* = 276, 27.21%), class II (*n* = 328, 32.34%), and class III (*n* = 410, 40.43%).

### 2.2 Cephalometric Parameters

All cephalometric parameters included in this study are summarized in Table S1 and Figure S1.

### 2.3 Inclusion and Exclusion

In this study, patients were diagnosed according to the Calculated_ANB, defined as Calculated_ANB = ANB angle‐ individualized ANB of Panagiotidis and Witt (1977).

### 2.4 The Inclusion Criteria

1. Orthodontic patients:
  - Skeletal class I (−1.0 < Calculated_ANB < 1.0)
  - Skeletal class II (Calculated_ANB > 1.0)
  - Skeletal class III (Calculated_ANB < −1.0)
2. Patients with pre‐treatment lateral cephalograms available.

### 2.5 The Exclusion Criteria

1. Patients who had no pre‐treatment records are available.

Although the ranges of the Calculated_ANB were set as mentioned above, the final diagnosis was done in accordance with the clinical diagnosis of the orthodontic team, and taking into account additional cephalometric parameters (e.g., ANB angle, and Wits appraisal). In this study, the majority of cases were aligned within the expected range: Skeletal class I (196 cases within the expected range, 71%), class II (304 cases within the expected range, 92%), and class III (402 cases within the expected range, 98%).

### 2.6 Data Analysis

Data analysis was performed using the Python software platform. In this study, we used linear discriminant analysis (LDA) (Xanthopoulos et al. 2013), random forest (RF) (Breiman 2001), decision tree (DT) (Podgorelec et al. 2002), K‐nearest neighbors (KNN) (Zhang et al. 2017), support vector machine (SVM) (Pisner and Schnyer 2020), and naive Bayes (NB) (Reddy et al. 2022) as classification models. All classifiers were implemented using the Scikit‐Learn Python package (Pedregosa et al. 2011). In addition, we calculated the parameters' importance using two techniques—the impurity decrease (Lin 2023), and the leave‐one‐feature‐out (LOFO) technique (Rakimbekuulu et al. 2024) using cross‐validation 10.

### 2.7 Artificial Neural Networks (ANNs)

An artificial neural network (ANN) is a mathematical model inspired by biological nervous systems like the human brain. ANN's ability to learn quickly enabled the use of this model efficiently for classification, pattern recognition, and modeling (Yenamandra et al. 2021; Ardizzone et al. 1988). In this study, we will use an ANN to classify the patients as skeletal class I, II, or III patterns based on the cephalometric parameters and covariates such as gender and age.

### 2.8 Data Validation

For the machine learning, the models were validated using the unseen set, which included 20%, and the accuracy, precision, recall, and F1‐score of each model were calculated. For the deep learning model, we validated all models using accuracy, with early stopping to ensure optimal generalization.

### 2.9 Sample Size

In this study, the sample size was decided according to the maximum available cases within the defined period of patient enrollment. Furthermore, the results of the machine‐learning and deep‐learning models were examined on the validation data (20% unseen data), and the results showed the ability of the current sample size to gain the desired results. Therefore, the final sample included 1014 patients divided into three skeletal classifications.

## 3 Results

### 3.1 Descriptive Statistics

The mean age of skeletal class I patients was 18 years (SD = 6.5), with an age range of 8.6–55. Among class I patients, females constituted more than half (*n* = 183,66.30%). Skeletal class II patients' mean age was 17 years (SD = 6.5), with an age range of 7–44, and here also, females were more than half of the patients in this class (*n* = 218,66.46%). Finally, in skeletal class III patients, the mean age of the patients was 18 years (SD = 7.7), with an age range of 4–54, and here also, females were more than half of the patients in this class (*n* = 211,51.46%). Table 1 summarizes the complete, detailed information about each class.

**Table 1:** Descriptive statistics of the study sample.

	Class: I			Class: II			Class: III		
Variable	*N*	Mean	Std. dev.	*N*	Mean	Std. dev.	*N*	Mean	Std. dev.
Females	183 (66.30%)			218 (66.46%)			211 (51.46%)		
Age	276	18	6.5	328	17	6.5	410	18	7.7
NL‐ML angle	276	29	6.1	328	28	6.7	410	29	6.6
NL‐NSL angle	276	8.4	3.5	328	8.2	3	410	7.8	3.5
PFH/AFH	276	66	5.8	328	65	5.4	410	65	5.5
Gonial angle	276	131	6.9	328	129	7.8	410	135	7.7
Facial axis	276	88	4.5	328	88	4.8	410	91	5.3
SNA angle	276	82	3.9	328	83	3.7	410	82	4.1
SNB angle	276	78	3.2	328	76	3.2	410	82	4.6
ANB angle	276	4.7	1.7	328	7.2	1.8	410	−0.38	2.9
ANB_ind_	276	5.2	1.5	328	5.2	1.7	410	4.9	1.6
Calculated_ANB (ANB – ANB_ind_)	276	−0.49	0.88	328	2	1	410	−5.2	2.4
SN‐Ba angle	276	129	5.5	328	130	5.3	410	127	5.6
SN‐Pg angle	276	78	3.6	328	76	3.6	410	82	4.8
S‐N (mm)	276	63	7.9	328	63	7.2	410	59	12
Go‐Me (mm)	276	61	7.6	328	59	6.8	410	60	13
Wits appraisal (mm)	276	0.86	2.7	328	5.2	3	410	−9.4	5.5
ML‐NSL angle	276	37	6.1	328	36	7.3	410	37	7.6
+1/NL angle	276	114	6	328	113	7.4	410	116	7.2
+1/SNL angle	276	106	6.9	328	105	8.2	410	108	7.8
+1/NA angle	276	23	6	328	22	7.8	410	27	6.7
+1/NA (mm)	276	3.8	2.1	328	3	2.4	410	4.3	2.5
−1/ML (anatomic)	276	93	7	328	96	8.8	410	86	7.6
−1/NB angle	276	28	6.3	328	28	6.9	410	25	6.8
−1/NB (mm)	276	5.6	2.3	328	5.6	2.5	410	4.8	3
Interincisal angle	276	125	9.7	328	123	11	410	128	11

*Note:* This table represents the descriptive statistics for each skeletal class: Sample size (N), mean, and standard deviation.

### 3.2 Machine‐Learning Algorithms

The machine‐learning models were evaluated by calculating the accuracy of each model on the unseen data, in addition to calculating the precision, the recall, and the f1 score.

### 3.3 General Machine‐Learning Models

The machine‐learning results demonstrated that the models could accurately predict the classification of the patients as skeletal class I, II, or III. When using all cephalometric parameters, in addition to gender and age as covariates, the machine‐learning models reached an accuracy of up to 99% in the random forest model. The random forest model demonstrated perfect precision among skeletal class III patients (precision = 100%), 98%, and 97% precision among skeletal class I and II, respectively. In addition, the results demonstrated an accuracy of 98% in the multi‐logistic model and the SVM models, followed by 96% in the decision tree model, 93% in the Gaussian naïve Bayes model, 91% in the LDA model, and 81% in the KNN model, as presented in Table 2.

**Table 2:** Machine learning models depend on the Calculated_ANB.

	Included parameters: 24 cephalometries and 2 covariates				Included parameters: Calculated_ANB (ANB – ANB_ind_)			Included parameters: Calculated_ANB and Wits appraisal		
	Class	Precision	Recall	F1score	Precision	Recall	F1score	Precision	Recall	F1score
Linear discriminant analysis	1	0.80	0.82	0.81	0.82	0.92	0.87	0.80	0.88	0.84
	2	0.88	0.94	0.91	0.94	0.99	0.96	0.92	0.99	0.95
	3	1.00	0.93	0.96	1.00	0.89	0.94	1.00	0.88	0.94
Accuracy				0.91			0.93			0.92
Random forest	1	0.98	0.96	0.97	—	—	—	—	—	—
	2	0.97	0.99	0.98	—	—	—	—	—	—
	3	1.00	1.00	1.00	—	—	—	—	—	—
Accuracy				0.99			**—**			**—**
Decision tree	1	0.86	0.98	0.92	0.81	0.88	0.85	0.92	0.88	0.90
	2	0.98	0.93	0.95	0.97	0.96	0.96	0.94	0.94	0.94
	3	1.00	0.96	0.98	0.95	0.92	0.93	0.98	1.00	0.99
Accuracy				0.96			0.92			0.95
K‐nearest neighbors	1	0.61	0.72	0.66	0.85	0.90	0.87	0.94	0.94	0.94
	2	0.80	0.82	0.81	0.96	0.94	0.95	0.96	1.00	0.98
	3	0.99	0.86	0.92	0.98	0.95	0.96	1.00	0.96	0.98
Accuracy				0.81			0.94			0.97
Support vector machine	1	0.92	0.98	0.95	0.88	0.92	0.90	0.90	0.92	0.91
	2	1.00	0.96	0.98	0.97	0.97	0.97	0.97	0.96	0.96
	3	0.99	0.99	0.99	0.98	0.95	0.96	0.98	0.98	0.98
Accuracy				0.98			0.95			0.96
Gaussian naive Bayes	1	0.82	0.92	0.87	0.90	0.90	0.90	0.96	0.90	0.93
	2	0.96	0.96	0.96	0.96	0.97	0.96	0.93	0.97	0.95
	3	0.97	0.91	0.94	0.96	0.95	0.96	0.99	0.99	0.99
Accuracy				0.93			0.95			0.96
Multi class logistic regression	1	0.94	0.96	0.95	0.90	0.92	0.91	0.94	0.88	0.91
	2	0.97	0.97	0.97	0.97	0.99	0.98	0.94	0.99	0.96
	3	1.00	0.99	0.99	0.98	0.95	0.96	0.98	0.98	0.98
Accuracy				0.98			0.96			0.96

*Note:* Applying different machine‐learning models to the studied cohort. The table presents the linear discriminant analysis, random forest, decision tree, K‐nearest neighbors, support vector machine, Gaussian naïve Bayes, and multi‐class logistic regression. For every model, we presented the precision, recall, F1 score, and accuracy.

### 3.4 Machine‐Learning Parameters Are Essential According to the Most Accurate General Model (the Random Forest Model)

As shown in the previous section, the most accurate model was the random forest, with an accuracy of 99%. To better understand the importance of each parameter included in the model, we calculated the importance of each parameter using the impurity decrease, as presented in Table 3A. The most important variable in the model was the Calculated_ANB, followed by the parameters' Wits appraisal, ANB angle, SNB angle, SNPg angle, and −1/ML, respectively.

**Table 3:** Cephalometric parameters importance based on the random forest model.

A.	
Feature	Importance
Calculated_ANB	0.34
Wits	0.22
ANB	0.15
SNB	0.04
SNPg	0.03
1MeGo	0.02
Gonial_angle	0.02
ML‐NSL	0.01
SNA	0.01
1i/NA mm	0.01
PFH/AFH	0.01
NL/ML	0.01
1/NB	0.01
ANBind	0.01
s‐n (mm)	0.01
Go‐Me(mm)	0.01
Age	0.01
Interincisal angle	0.01
1/NA	0.01
NL/NSL	0.01
Facial axis	0.01
1/NL	0.01
1i/NB MM	0.01
SN‐Ba	0.01
1/SN	0.01
Gender01	0.00

B.		
Feature	Mean	Standard deviation
Calculated_ANB	0.10	0.12
Wits	0.08	0.19
NL/ML	0.07	0.15
NL/NSL	0.07	0.15
ANB	0.06	0.14
Facial axis	0.06	0.14
s‐n (mm)	0.06	0.15
Age	0.06	0.15
PFH/AFH	0.06	0.15
ANBind	0.05	0.16
1MeGo	0.05	0.16
SNPg	0.05	0.15
1/NA	0.05	0.14
1/NB	0.05	0.15
SNA	0.05	0.16
SN‐Ba	0.05	0.15
SNB	0.05	0.13
1i/NA mm	0.04	0.06
1/SN	0.04	0.06
ML‐NSL	0.04	0.07
Gender01	0.04	0.07
1i/NB MM	0.04	0.07
Gonial_angle	0.04	0.06
1/NL	0.03	0.07
Interincisal angle	0.03	0.07
Go‐Me(mm)	0.03	0.06

*Note:* This table presents the importance of the cephalometric parameters to classify an individual as skeletal class I, II, or III through the random forest machine‐learning model. This table was extracted using the classifier. feature_importances_ function in Python. B—Cephalometric parameters importance based on leave‐one‐feature‐out (LOFO) technique. This table presents the feature importance using leave‐one‐feature‐out (LOFO) technique and includes the mean and standard deviation of the accuracy decreases for each parameter.

### 3.5 Feature Importance Using Leave‐One‐Out (LOFO) Technique

For a better understanding of the importance of each parameter, we performed leave‐one‐feature‐out (LOFO) technique, which leaves one variable out of the random forest, every time. As expected, the Calculated_ANB was also the most important variable, and removing it will cause almost a 10% decrease in accuracy. The following variables were the Wits appraisal, NL/ML, NL/NSL, and ANB angles. Finally, the results showed that age and gender were placed in 8th, and 21st places (out of 26 variables), as described in Table 3B.

### 3.6 Stepwise Forward Machine‐Learning Models

**Figure 1:**
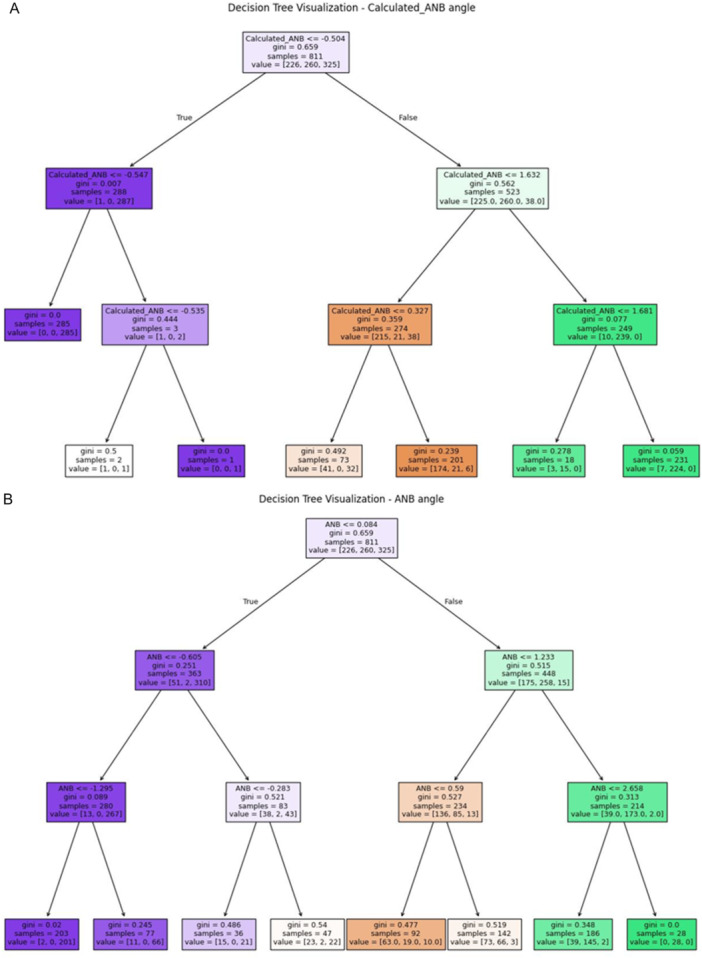
A, B: Figure 1A—Decision tree model based on the Calculated_ANB. This table presents the decision tree for the Calculated_ANB up to a maximum of three levels. Each node is labeled with the value of the Calculated_ANB. In addition, in each node, we have the number of patients in each class (i.e., skeletal class I, II, III), and the Gini score of purity (between 0 and 1). Figure 1B—Decision tree model based on the ANB angle. This table presents the decision tree for the ANB angle.

− Calculated_ANB as a single predictor
  The results demonstrated the remarkable ability of the Calculated_ANB only as a predictor to classify the patients as skeletal class I, II, or III, with an accuracy up to 96% in the multi‐class logistic model, followed by the SVM, and Gaussian naïve Bayes, which showed an accuracy of 95%. Besides, the KNN model demonstrated an accuracy of 94%, followed by 93% accuracy in the LDA model, and 92% in the decision tree model (Table 2). In addition, and in order to understand the decision tree and the Calculated_ANB angle limits that were set to classify the patients, we visualized the Calculated_ANB angle decision tree in Figure 1A.
− Calculated_ANB and Wits appraisal parameters
  In the next step, we added the Wits appraisal parameter and the Calculated_ANB to the machine‐learning models. The results showed an improvement in the accuracy of the KNN model to 97%, the SVM, and the Gaussian naïve Bayes to 96%, and finally, the decision tree model improved to 95%, as shown in Table 2.
  Finally, in this section, we applied the same machine‐learning models while adding the ANB angle to the Calculated_ANB and Wits appraisal; however, this did not improve validation accuracy in any of our models.

### 3.7 Classification Accuracy From the ANB Angle (i.e., Without the Calculated_ANB)

− ANB angle as a single predictor
  The results demonstrated the ability of the ANB only as a predictor to classify the patients as skeletal class I, II, or III, with an accuracy of up to 81% in the LDA model, followed by 80% accuracy in the KNN and multi‐class logistic models. Besides, the decision tree model, SVM, and Gaussian naïve Bayes models gained 79% accuracy. Full results are described in Table 4. In addition, and in order to understand the decision tree and the ANB angle limits set to classify the patients, we visualized the ANB angle decision tree in Figure 1B.
− ANB angle and Wits appraisal
  According to the random forest general model, the Wits appraisal was the most important after the Calculated_ANB, so here the addition of the Wits appraisal, to the ANB angle, resulted in an improvement in the results and the accuracy of the models, up to 91% accuracy in the Gaussian naïve Bayes model, followed by the LDA, SVM, and multi logistic models that gained accuracy 90%, and finally the decision tree and KNN models with 89% accuracy (Table 4).
− ANB angle, Wits appraisal, and SNB angle
  In this section, we added the SNB angle to the previous model and received a slight increase in the accuracy of part of the models; for example, the Gaussian naïve Bayes increased the accuracy from 91% to 92%. In addition, the model's SVM and multi‐class logistic regression increased from 90% to 91% accuracy, as detailed in Table 4.

**Table 4:** Machine learning models, while excluding the Calculated_ANB.

		Included parameters: ANB angle only			Included parameters: ANB angle and Wits appraisal			Included parameters: ANB angle, Wits appraisal, and SNB angle			Included parameters: Wits appraisal, SNA, SNB, and ML‐NSL angles		
	Class	Precision	Recall	F1score	Precision	Recall	F1score	Precision	Recall	F1score	Precision	Recall	F1score
Linear discriminant analysis	1	0.60	0.64	0.62	0.79	0.82	0.80	0.75	0.86	0.80	0.82	0.89	0.85
	2	0.82	0.87	0.84	0.86	0.91	0.89	0.89	0.93	0.91	0.90	0.95	0.93
	3	0.94	0.86	0.90	1.00	0.93	0.96	1.00	0.88	0.94	1.00	0.92	0.96
Accuracy				0.81			0.90			0.89			0.92
Random forest	1	—	—	—	—	—	—	0.81	0.78	0.80	0.88	0.87	0.88
	2	—	—	—	—	—	—	0.85	0.94	0.90	0.89	0.95	0.92
	3	—	—	—	—	—	—	1.00	0.94	0.94	1.00	0.97	0.98
Accuracy				**—**			**—**			0.90			0.94
Decision tree	1	0.57	0.64	0.60	0.80	0.78	0.79	0.75	0.78	0.76	0.87	0.89	0.88
	2	0.86	0.81	0.83	0.84	0.93	0.88	0.84	0.94	0.89	0.90	0.95	0.93
	3	0.89	0.87	0.88	1.00	0.93	0.96	1.00	0.88	0.94	1.00	0.96	0.98
Accuracy				0.79			0.89			0.88			0.94
K‐nearest neighbors	1	0.62	0.52	0.57	0.78	0.78	0.78	0.79	0.84	0.82	0.88	0.85	0.87
	2	0.80	0.88	0.84	0.86	0.90	0.88	0.87	0.90	0.88	0.89	0.95	0.92
	3	0.90	0.91	0.90	0.98	0.94	0.96	1.00	0.94	0.97	0.99	0.97	0.98
Accuracy				0.80			0.89			0.90			0.93
Support vector machine	1	0.58	0.58	0.58	0.83	0.76	0.79	0.82	0.82	0.82	0.88	0.85	0.87
	2	0.83	0.85	0.84	0.85	0.91	0.88	0.88	0.93	0.90	0.90	0.93	0.92
	3	0.89	0.87	0.88	0.98	0.96	0.97	0.99	0.94	0.96	0.98	0.98	0.98
Accuracy				0.79			0.90			0.91			0.93
Gaussian naive Bayes	1	0.58	0.58	0.58	0.83	0.80	0.82	0.83	0.88	0.85	0.87	0.87	0.87
	2	0.83	0.85	0.84	0.87	0.91	0.89	0.90	0.93	0.91	0.90	0.93	0.92
	3	0.89	0.87	0.88	0.98	0.96	0.97	1.00	0.94	0.97	0.99	0.97	0.98
Accuracy				0.79			0.91			0.92			0.93
Multi‐class logistic regression	1	0.59	0.58	0.59	0.82	0.80	0.81	0.82	0.84	0.83	0.90	0.89	0.90
	2	0.83	0.85	0.84	0.87	0.91	0.89	0.90	0.93	0.91	0.92	0.98	0.95
	3	0.89	0.88	0.89	0.98	0.95	0.96	0.98	0.94	0.96	0.99	0.96	0.97
Accuracy				0.80			0.90			0.91			0.95

*Note:* Different machine‐learning models were applied to the study data. The table presents the linear discriminant analysis, random forest, decision tree, K‐nearest neighbors, support vector machine, Gaussian naïve Bayes, and multi‐class logistic regression. For every model, we presented the precision, recall, F1 score, and accuracy.

### 3.8 Machine‐Learning Models Based on the Wits Appraisal and the Parameters That Define the ANB Angle and ANB_ind_

In this part, we decided to include in the machine‐learning models the most powerful parameters according to the previous models, and according to the parameters that are taken into account to calculate the ANB and ANB_ind_. In this section, we included the Wits appraisal, the SNA, SNB, and ML‐NSL angle. The results of this section demonstrated the ability of the multi‐class logistic regression to accurately classify the patients with 95% accuracy. The random forest and decision tree were with 94% accuracy, while the KNN, SVM, and Gaussian naïve Bayes were with 93% accuracy. Finally, the LDA model had 92% accuracy (Table 4).

### 3.9 Deep‐Learning Algorithms

#### 3.9.1 General Deep‐Learning Model

**Figure 2:**
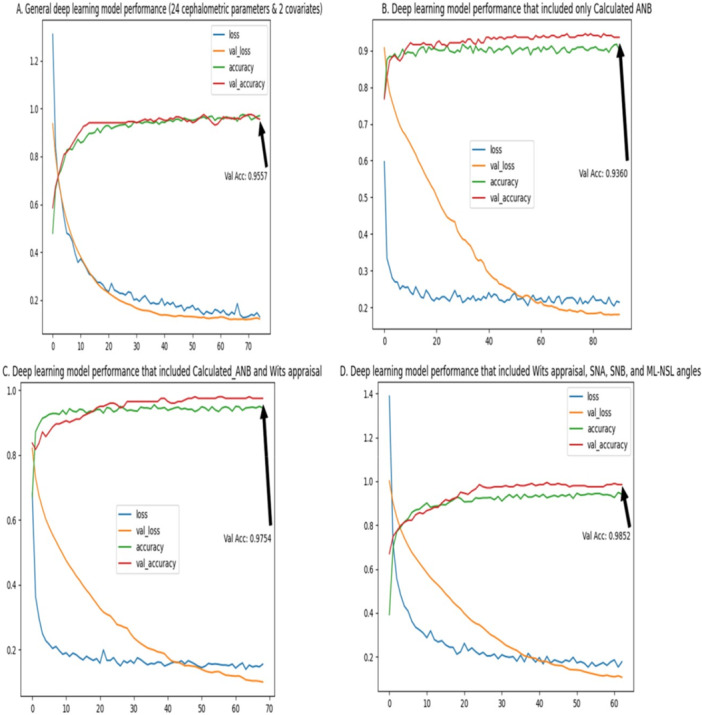
A–D: Deep learning models’ performance based on a different set of input parameters. In these figures, we present the performance of the deep‐learning models to classify the patients as skeletal class I, II, or III. For each model we present, we show the training loss function (the error margin between the prediction and the actual value), the validation loss, the training accuracy, and the validation accuracy. In each model, we used different input variables. In Figure 2A, we used all cephalometric variables, gender, and age. In Figure 2B, we used the Calculated_ANB only. In Figure 2C, we used the Calculated_ANB and Wits appraisal. Finally, in Figure 2D, we used the Wits appraisal, SNA, SNB, and ML‐NSL.

The results of the general deep‐learning model that included all cephalometric parameters and the covariates gender and age demonstrated the ability of this model to accurately classify the patients as skeletal class I, II, or III, with 95.5% accuracy in the validation (Training‐accuracy = 96.8%, Validation‐accuracy=95.5%) (Figure 2A).

#### 3.9.2 Stepwise Forward Deep‐Learning Models

− Deep‐learning model using Calculated_ANB only
  The results of the deep‐learning model that included only the Calculated_ANB as a predictor demonstrated only 93.6% validation accuracy (training‐accuracy=90.26%, validation‐accuracy=93.6%) (Figure 2B).
− Deep‐learning model using Calculated_ANB, and Wits appraisal
  Adding the Wits appraisal to the deep‐learning model significantly improved the validation accuracy of the model to 97.54% (training‐accuracy=94.18%, validation‐accuracy=97.54%) (Figure 2C).
  Finally, in this section, we applied a deep‐learning model while adding the ANB angle to the Calculated_ANB and Wits appraisal, but unfortunately, this didn't improve the validation accuracy.
− Deep‐learning models based on the Wits appraisal and the parameters that define the ANB angle and ANB_ind_
  Our results showed almost perfect accuracy in classifying the patients as skeletal class I, II, or III when applying a deep‐learning model that includes Wits appraisal, SNA, SNB, and ML‐NSL angles. The results showed that the validation accuracy is 98.52% (training‐accuracy=94.46%, validation‐accuracy=98.52%). The model performance is fully described in Figure 2D.

## 4 Discussion

The main aim of this study was to establish a novel artificial intelligence method in order to achieve as high an accuracy as possible in the classification of orthodontic patients into skeletal class I, II, or III. According to our results, the machine‐learning models were able to classify the patients accurately with an accuracy of 99% when using all the 24 cephalometric parameters, and gender and age as covariates. Besides, the deep‐learning model based on the Wits appraisal and the parameters that define the ANB angle and ANBind were able to classify the patients with an accuracy of 98.52%, while the machine‐learning model was able to classify the patients with an accuracy of up to 95% using the same inputs. According to the individualized ANB that was established by Panagiotidis and Witt (1977), the ability of the regression formula to correctly classify the patients as skeletal class I, II, or III was limited, with an *R*2 = 0.8, which means that around 20% of the cases will be missed as outliers.

In a previous study, Zhou et al. (2023) proposed a craniofacial machine‐learning diagnostic workflow using 408 X‐ray lateral cephalograms from Chinese patients from 2017 to 2021. It demonstrated that the deep‐learning model multi‐layer perceptron achieved 97.56% accuracy for sagittal diagnosis. Our current results demonstrated higher performance and accuracy than previously proposed models. In another study that aimed to establish an automated skeletal classification based on 5890 lateral cephalometric images from Ewha Woman's University Medical Center, and used a convolutional neural network (CNN), found that the sensitivity, specificity, and accuracy for vertical and sagittal skeletal diagnosis were >90% (Yu et al. 2020). In another research that was done on a patients who attended the health center in the Orthodontist Division, University of Peradeniya, that included 60 subjects, and proposed a classification model to predict the malocclusion patterns of orthodontic patients, found that the accuracy of the multinomial logistic regression model, k‐NN algorithm, random forest, and Naïve Bayes classification of malocclusion patterns were 88.89%, 83.33%, 88.89%, and 55.56%, respectively (Jayathilake et al. 2021). Furthermore, the current models included all skeletal classifications (i.e., skeletal class I, II, III), in contrary to the previously published studies, which applied machine‐learning models to classify skeletal class II and III only (Midlej et al. 2024), and another study that was performed on skeletal class I and II Arab patients and gained only 0.87 accuracy (Midlej et al. 2025).

In this study, the general model of the random forest model was the best model with the best accuracy performance. In the random forest model, we calculated the importance of each parameter using the impurity decrease (Table 3A), and then used the LOFO technique and performed multiple times, while each time keeping one feature out. In both techniques, the Calculated_ANB was the most important feature, followed by the Wits appraisal. These results are supported by many studies that demonstrated the ability of the individualized ANB and the Wits appraisal to explain the variety of facial types. In a study that was performed by Yen (1990), the individualized ANB angle of Chinese adults was examined, and it was found that the ANB angle for different facial types is presented through equation‐produced lists of individualized norms. These norms aid the interpretation of the individual variations. In another study that was performed by Zamora et al. (2013), and analyzed the ANB and Wits values and their relationship with other measurements in diagnosing the anteroposterior maxillo‐mandibular relationship using CBCT, concluded that ANB and Wits appraisal must be included in 3D cephalometric analyses as both are necessary to undertake a more accurate diagnosis of the maxillo‐mandibular relationship of the patients. In addition, the LOFO method demonstrated that age was the 8th important variable, while gender was the 21st. In the study that was performed by Zamora et al. (2013), it was found that no correlation between either Wits or ANB in relation to the age of the individuals. In another study that was performed by Bishara et al. (1983), the ANB angle changes significantly with age, while the Wits appraisal indicates that the relationship between points A and B does not change significantly with age. Regarding gender dimorphism variations, previous studies have shown significant variations between males and females in malocclusion phenotypes. For example, the study of Baccetti et al. (2005), which examined gender differences among skeletal class III patients, found that male subjects with class III malocclusion present with significantly larger linear dimensions of the maxilla, mandible, and anterior facial heights. In another study that examined gender differences in lower facial soft tissue thickness among different skeletal patterns, found that in class I, class II division 1, class II division 2, and class III malocclusions, males demonstrated a significant difference in lower soft tissue thickness (characterized as thicker lower facial soft tissue) compared to female patients (Alhumadi et al. 2022). In Figure 1A,B, the results showed that the decision tree was able to classify the patients as skeletal class III, mainly if the Calculated_ANB was lower than −0.54, and as skeletal class II if they are higher than 1.63. Regarding the ANB angle, the results showed that the root of the tree was basically divided if the patient had less than 0.084 degrees, and then the patient is probably diagnosed as skeletal class III, and if the patient had more than 1.23 degrees, then in most cases, he will be skeletal class II. These limits and ranges emphasize the variations between the different ethnic groups. According to Steiner (1953), the ANB angle between 0 and 4 is the normal occlusion (skeletal class I), the ANB angle with values > 4° means the patient is skeletal class II, and the ANB angle with values < 0° means the patient is skeletal class III. This definition was adopted by many orthodontists and researchers (Alghamdi and Tashkandi 2022; Lombardo et al. 2012).

### 4.1 Limitations

First, in this study, we included only a single ethnic group, and the models that were applied in this study may be less accurate for other ethnic groups. One more limitation of this study was the moderately low sample size, and future research should include more cases. In summary, addressing these two points in future research can contribute to the generalizability of the mentioned models in this paper. Finally, in this research, we recommend that future research will not only take the traditional cephalometric parameters, but also apply AI models for landmark detection and improved diagnosis.

## 5 Conclusions and Clinical Impact

This study emphasizes the ability of machine‐learning and deep‐learning algorithms to accurately classify patients as skeletal class I, II, or III. The results of this study overcome the limitations of the typical classification, that can't classify the patient accurately. One of the impressive models presented in this study is the model that includes only the parameters Wits appraisal, SNB, SNA, and ML‐NSL, and can predict and classify the patients with 98% accuracy. This model will enable the orthodontist to easily classify patients without the need for any additional equations. In addition, we applied leave‐one‐feature‐out technique (LOFO) for multiple random forest models and found that the Calculated_ANB (ANB angle‐individualized ANB), and Wits appraisal were the most important parameters in the random forest models. Besides, age and gender were in 8th and 21st places (out of 26 variables). Furthermore, the decision tree results demonstrated the distinct characteristics of this ethnic group, which were presented by different ranges of ANB angles that define every skeletal class. The results showed that the tree's root was divided if the patient had less than 0.084 degrees, then the patient was probably diagnosed as skeletal class III, and skeletal class II If he/she is higher than 1.23. In summary, the models used in this research are one more step towards the use of automated classification of skeletal malocclusion, using AI methods, and thus contribute to more precise diagnosis and treatment.

## Author Contributions

Kareem Midlej contributed to design, data acquisition, and analysis, as well as drafted the manuscript. Peter Proff contributed to conception and design, data acquisition, and critically revised the manuscript. Nezar Watted contributed to conception, data acquisition, and analysis, and critically revised the manuscript. Fuad A. Iraqi contributed to conception and design, data acquisition, analysis, and interpretation, supervised the project, and critically revised the manuscript. All authors gave their final approval and agreed to be accountable for all aspects of the work.

## Ethics Statement

According to current guidelines and following the Ethics Committee of Tel‐Aviv University, ethics and regulations. The committee reviewed and approved this research project and study design with approval number 0010557‐4.

## Consent

Informed consent was obtained from all subjects involved in the study.

## Conflicts of Interest

The authors declare no conflicts of interest.

## Supporting information

Supporting File

## Data Availability

The data that support the findings of this study are available from the corresponding author upon reasonable request.
