# Supplementary material for: Applying Automated Artificial Intelligence Models on Lateral Cephalometric Parameters to Accurately Classify Arab Orthodontic Patient Patterns
Source: Clin Exp Dent Res. 2026 Jun 7;12(3):e70372. doi: 10.1002/cre2.70372 (PMC13242863; doi:10.1002/cre2.70372)
Supplement: Supplementary file 1 — Supporting File [file CRE2-12-e70372-s001.docx]

| **Dimension/Group** | **Parameter** | **Unit** | **Definition** |
| --- | --- | --- | --- |
| Vertical Analysis | NL/ML (anatomic) | ° | The angle between the NL and ML |
| Vertical Analysis | SNL/ML (anatomic) | ° | The inclination of the mandible (mandibular inclination) relative to the nasion-sella line (anterior skull base, SNL) |
| Vertical Analysis | NL/NSL | ° | The angle between Sella-Nasion-line (NSL = SN) and nasal line (Spa-Spp) |
| Vertical Analysis | PFH/AFH | % | The ratio between posterior (SGo) and anterior (NMe) facial height |
| Vertical Analysis | Gonial Angle | ° | The angle between ML and line GoAr at Gonion |
| Vertical Analysis | Facial axis | ° | The angle between the lines NBa and PtGN’ |
| Sagittal Analysis | Angle SNA | ° | The angle between Sella, Nasion, and point A |
| Sagittal Analysis | Angle SNB | ° | The angle between Sella, Nasion, and point B |
| Sagittal Analysis | ANB | ° | The angle between Nasion, point A, and point B |
| Sagittal Analysis | ANB_ind_ | ° | ANB_ind_ = (−35.16 + 0.4 · SNA + 0.2 · ML-NSL) according to Panagiotidis and Witt |
| Sagittal Analysis | Calculated_ANB |  | ANB - ANB_ind_ |
| Sagittal Analysis | SN-Ba | ° | Central saddle angle. It describes the extent of the skull base flexion |
| Sagittal Analysis | SNPg | ° | The angle between Sella, Nasion and Pogonion |
| Sagittal Analysis | S-N | mm | The S-N line represents the anterior cranial base. It is constructed by connecting the points sella turcica and the Nasion |
| Sagittal Analysis | Go-Me | mm | The mandibular plane as a line connecting the points gonion and menton |
| Sagittal Analysis | Wits | mm | This parameter measures the extent to which the jaws are related to each other anteroposteriorly. |
| Growth Analysis | ML-NSL | ° | The angle formed between the ML and NSL lines |
| Dental Analysis | (+1/NL) | ° | The angle between upper incisors’ tooth axis and line NL |
| Dental Analysis | (+1/SN) | ° | The angle between upper incisors’ tooth axis and line SN |
| Dental Analysis | +1/NA | ° | The angle between upper incisors’ tooth axis and line NA |
| Dental Analysis | +1/NA | mm | The perpendicular distance between the upper central incisor to N-A provides information about the sagittal position of the incisor teeth. |
| Dental Analysis | -1/ML (anatomic) | ° | The relative anteroposterior angulation of the lower incisor teeth is determined by relating the most protruding incisor tooth to the mandibular plane (ML) |
| Dental Analysis | (-1/NB) | ° | The lower central incisor to N-B indicates the axial inclination of these teeth |
| Dental Analysis | (-1/NB) | mm | The perpendicular distance between the lower central incisor to N-B provides information about the sagittal position of the incisor teeth. |
| Dental Analysis | Interincisal angle | ° | The inter-incisal angle relates the inclination of the upper incisor to that of the lower incisor |

**Supplementary Table 1.** Cephalometric parameters definitions – this table represents the cephalometric parameters that were extracted from the lateral cephalogram image, and were included in the AI models

**Supplementary Figure 1. Lateral cephalogram parameters**


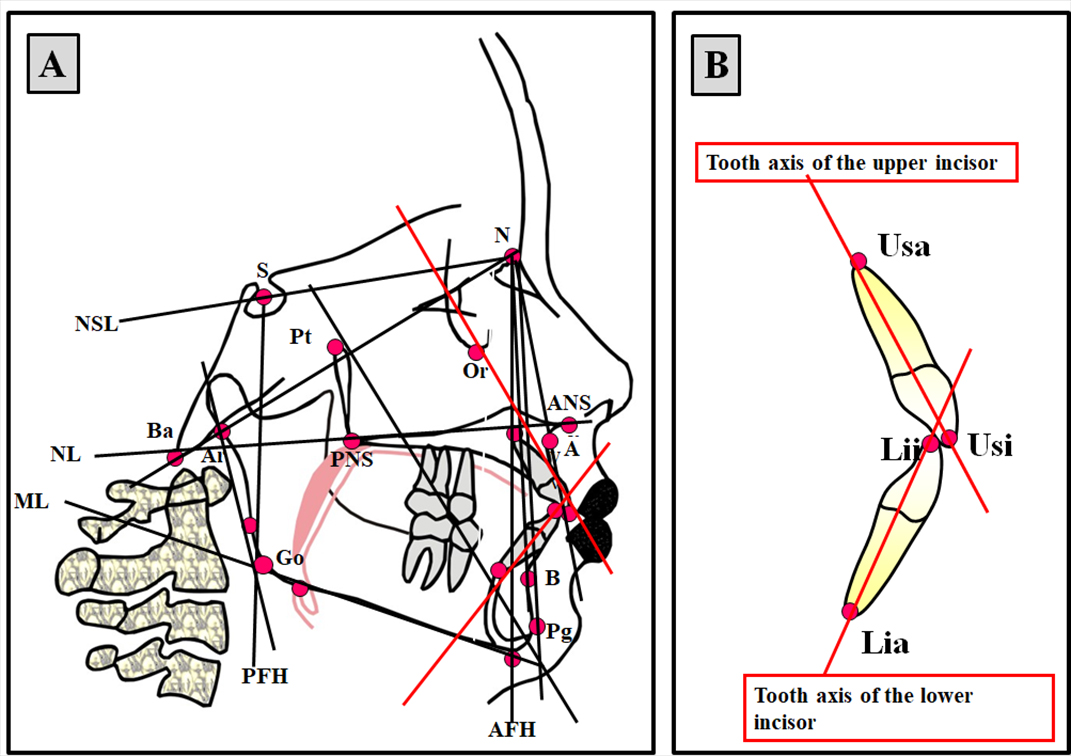


**Appendix Figure 1:** Represents the most crucial points extracted from the lateral cephalogram in this study (**Figure 1A**). Among these points are Nasion (N), Sella (S), Pterygoid point (Pt), Basion (Ba), Orbitale (Or), Subspinale (“A” Point), Supramentale (“B” point), and other points. **Figure 1B** shows the tooth axis of the upper and lower incisors positions and angles.
